# Between a rock and a hard place: Ethics, nurses' safety, and the right to protest during the COVID‐19 pandemic

**DOI:** 10.1111/inr.12703

**Published:** 2021-09-22

**Authors:** Fhumulani Mavis Mulaudzi, Mutondi Mulaudzi, Rafiat Ajoke Anokwuru, Mashudu Davhana‐Maselesele

**Affiliations:** ^1^ Department of Nursing University of Pretoria Pretoria South Africa; ^2^ Department of Public, Constitutional and International Law University of South Africa Pretoria South Africa; ^3^ Department of Nursing University of Pretoria Pretoria South Africa; ^4^ Department of Nursing Walter Sisulu University Mthatha South Africa

**Keywords:** COVID‐19, essential services, ethics, nurses’ rights, pandemic, patient's rights, personal protective equipment, right to strike, Safe environment, Ubuntu

## Abstract

**Aim:**

In this paper, we critically discuss the ethics of nurses' choice to strike during the COVID‐19 pandemic, considering legal and ethical arguments, overlaying the Ubuntu philosophy, an African ethic.

**Background:**

The recent unprecedented coronavirus disease pandemic and the increased reports on the absence of personal protective equipment in South Africa places many health workers' lives at risk. Nurses spend most of their time with patients, which exposes them to fatal risks as they work in unsafe environments.

**Research Methods:**

Exploratory literature review was conducted using Pubmed, CINAHL, Google Scholar and Science Direct) and law cases repository.

**Findings:**

Nurses thus may be justified in striking to protect their safety. State healthcare entities are obliged to ensure safety and protect the health of professionals during the pandemic. According to their Code of Practice and Pledge of Service, they are ethically obliged to put patients first, and as a result, they are legally barred from engaging in strike action.

**Conclusion:**

We conclude that there may be constitutional human rights arguments to support strike action. We also find that ethical principles alone do not provide clear direction to guide nurses in making justified and ethical decisions regarding service provision in an environment threatening to compromise their safety.

## INTRODUCTION

In December 2019, the coronavirus disease (COVID‐19) broke out in Wuhan, China, entering the global public health arena, causing great havoc to health systems in developed and developing countries (Liu et al., 2020). According to the World Health Organization (WHO) on the 6 July 2021, globally 183 934 913 people had been infected and 3 985 022 had lost their lives (WHO, 2021). In South Africa, over 2 million people have been infected with 62 171 people dead from the viruses with nurses and other healthcare workers included (WHO, 2021).

In March 2020, WHO declared the disease a pandemic and recommended measures such as regular sanitization, hand washing, personal protective equipment (PPE), and social distancing to combat the spread of the virus (Cucinotta & Vanelli, 2020). The sudden and devastating virus has exposed the inadequacies of the healthcare systems to manage massive public infections such as COVID‐19 (Gilbert et al., 2020; Gondi et al., 2020). Since the declaration of the pandemic, there have been first, second, and currently third waves of the virus. These waves refer to the sustained rise and fall in the number of infections at different times (Zhang et al., 2021).

Due to the suddenness of the first wave, healthcare systems worldwide experienced shortages of PPE, which protects healthcare workers from getting infected through exposure to infected patients, subsequently spreading the infection to seemingly healthy patients (Ip et al., 2020; Rowan & Laffey, 2020). The situation has exposed the readiness and willingness of the employers to provide a conducive and safe environment for health workers, consonant with their fiduciary labor relations responsibilities. At the same time, the issue of workers' rights to work in a safe environment and exercise these rights has also gained traction (McQuoid‐Mason, 2020).

In a bid to ensure a safe working environment, the WHO called for rationed use of PPE and provision of suitable PPE to healthcare workers, including nurses, as stated below:

“Provide adequate Infection Prevention and Control (IPC) and PPE supplies (masks, gloves, goggles, gowns, hand sanitizer, soap and water, cleaning supplies) in sufficient quantity to healthcare or other staff caring for suspected or confirmed COVID‐19 cases” (WHO, 2020).

The same call for rationed IPC and PPE use was echoed by the National Department of Health in South Africa in the following directive aimed at combating the spread of COVID‐19:

“All health authorities should endeavor to source and provide health equipment, sanitation material and medical supplies to various sites as may be required to respond to the COVID ‐19 outbreak” (Department of Health, 2020).

According to these directives, employers are responsible for providing all health workers with PPE, training, risk allowance, and reimbursement in exposure to occupational hazards. The International Labor Organization (ILO) states that employers are responsible for ensuring a safe working environment for employees and having enough resources to render quality nursing care. In South Africa, the Occupational Health and Safety Act (No. 181 of 1993 as amended) protects workers’ well‐being by ensuring their uncompromised safety from any work‐related hazards (Department of Labor, 1993). It is expected that during the first wave, the healthcare system would have made amendments and made provisions to delay the spread while preparing resources to fight the scourge. Unfortunately, this was not necessarily the case. Hospitals still experienced a shortage of PPE, exposing the healthcare workers more to the risk of infection, fatigue, and mental exhaustion (Chersich et al., 2020).

In the race against reducing the transmission rate, at the end of the first wave, vaccines were rolled out globally. Vaccination of healthcare workers and the general populace was done in many developed countries to control the surge of infections (Hall et al., 2021). However, the roll‐out of vaccination in South Africa has been slow and has attracted hesitancy among the public (Dzinamarira et al., 2021). This slow roll‐out has further exposed healthcare workers to unsafe working environments, making the third wave deadlier.

As frontline workers in the battle against COVID‐19, nurses have the right to work in safe environments (McQuoid‐Mason, 2020), although this is not often the reality on the ground. Nurses around the world have expressed concerns over the lack of PPE in COVID‐19 designated hospitals. In most cases, employers failed to adhere to the standards of care and protection prescribed by WHO, resulting in frontline workers being exposed to infection (McQuoid‐Mason, 2020). Padilla (2020), for example, documented nurses calling for public assistance to provide masks because their employers expected them to wear one mask for 5 days, increasing their risk of infection.

The failure of the State and the healthcare systems to create safe environments for healthcare workers during the COVID‐19 pandemic has led to a spate of outcries from nursing organizations throughout the world. For example, the American Nurses Association, the Royal College of Nursing, and the International Council of Nurses (ICN) have all registered their dissatisfaction with the scant supply of PPE and the subsequent risk it poses to nurses as frontline healthcare workers (American Nurses Association, 2020; Kennedy, 2021; Royal College of Nursing, 2020). Similarly, nurse unions in South Africa vehemently voiced their dissatisfaction by threatening not to touch any patients if they are not provided with PPE (Mathe, 2020). One of the unions even took the State to court while other unions have threatened to embark on strike action (Roelf & Winning, 2020). Chersich et al. (2020) maintain that African healthcare workers must raise their voices regarding their needs for a safe working environment, including the provision of PPE, to inform policies during the COVID‐19 pandemic.

Strike actions are often embarked on by aggrieved workers when their labor rights are not upheld (Gernigon et al., 2000; Novitz, 2016). When it comes to strike action, professional nurses have sometimes been publicly criticized. Nurses have often been viewed as breaching their oaths and behaving unethically if they put their rights above those of the patient (Fisher, 2017). Often this raises the question: *Are nurses entitled to human rights?* Nurses, first, are human before being nurses. Therefore, nurses are subject to give and receive fundamental ethics contained in the human rights charter (Haddad & Geiger, 2020; Oguisso et al., 2019). Nursing has always been an indispensable pillar of healthcare systems; their action or inaction greatly determines patient health outcomes. Nurses are expected to deliver quality healthcare services to patients in healthy and safe environments as clearly articulated in the Patients' Rights Charter (Daniels, 2020; Singh & Mathuray, 2018).

The overarching questions that this paper seeks to answer are “*Whether strike action by nurses as frontline workers during a pandemic is ethical? What are the different principles of ethics that must be considered when deciding to protest?”* This paper considers five main principles of ethical decision‐making that should be considered before embarking on strike action.

## ETHICAL DECISION‐MAKING

During this COVID‐19 pandemic, the ethical decision‐making skills of a nurse are critical. Ethical decision‐making is the process of making decisions based on ethical principles when faced with an ethical dilemma (Roshanzadeh et al., 2020). Nurses have the unacceptable choice between caring for the lives of their patients and risks to their own lives in an environment without PPE. Ethical decision‐making models could assist nurses in making choices imbued with moral courage, which relates to the strength of character to adhere unwaveringly to one's beliefs and sense of values and morals. In some instances, moral courage becomes the propellant for protecting ethical decision‐making to resist authoritative behavior by the State as an employer.

Moral courage also signifies the ability of professionals to persevere and stand up for what is right irrespective of support from others, especially on life‐and‐death matters (Murray, 2010). Similarly, in the COVID‐19 context, moral courage is required to inform nurses' decisions on whether to treat and care for a patient in the absence of PPE. A nurse who feels strongly about the right to work in a safe and healthy environment will necessarily require moral courage to refuse to administer care without PPE. Likewise, a nurse who morally and professionally decides to care for a patient despite the availability of PPE still requires moral courage to stand by their decision. However, moral courage should not be confused with moral arrogance and moral certitude. Moral certitude fortifies the conviction of individuals about the absolute correctness of their decisions. Meanwhile, moral arrogance depicts the condescension with which the opposing choices, views, and decisions are undermined (Murray, 2010). In a situation where group dynamics loom large, the moral courage of an individual could be arrogantly subsumed by the collective group and morality paraded as that particular group's identity (Mwiandi, 2011). For instance, the moral certitude or individual choice or decision of a nurse belonging to a group (e.g., labor union) could be challenging to express in a situation where the dominant views and decisions of the majority prevail unilaterally.

In some instances, ethical principles tend to contradict each other, requiring intuition as the primary factor for balancing and resolving such contradictions. Accordingly, nurses may resort to ethical principles to make informed decisions on whether to participate in a strike or withhold their (essential) services to patients on account of the employer failing to provide PPE. However, there may be conflicting ethical principles since they (principles) do not necessarily conform to a rank‐ordered or hierarchical application, thus making it challenging to balance beneficence against autonomy during decision‐making moments.

In the current COVID‐19 context, the nurse is then confronted with deciding the dilemma: *“Do I prioritize doing good by taking care of patients despite the possibility of me contracting the virus?”* This has no clear‐cut answer. Furthermore, during nursing practice in the pandemic, there is often no exhaustive account using principles as a framework for moral reasoning. In addition, the principles do not provide a full philosophical justification for decision‐making. It then becomes difficult to make an informed decision, as ethical decision‐making models only provide guidelines but not proper answers (Page, 2012).

### Professional and moral obligation during a pandemic

Nurses are professionally and morally obliged to take care of patients (Dowie, 2017). Such obligation extends to caring for patients during a health emergency like a pandemic and risky situation such as war and natural disasters (Leider et al., 2017). Professional obligation is embodied in ethical codes of nursing and the oath that nurses take before they start to practice as health professionals. In many countries, when taking their oath, nurses declare to care for patients without considering their creed, gender, or nationality. Most importantly, nurses declare in their oath that patients will be their first consideration. The oath is declared in front of their parents and members of the public as witnesses. Nurses are thus obliged to promote health, prevent disease, and alleviate the suffering of individual patients (ANA, 2019). However, fulfilling these oaths becomes a “gray area” when nurses must balance their own safety and that of patients. Similar concerns with the dilemma of obligations facing healthcare professionals have also been expressed by Chima (2013), who asserts that doctors and other healthcare workers have a fiduciary obligation to patients.

Nurses in South Africa are also governed by and obligated to the laws of the land. These include The Constitution of the Republic of South Africa (Act No. 108 of 1996), the Labor Relations Act (LRA; No. 66 of 1995), the Basic Conditions of Employment Act (No. 75 of 1997), and the Nursing Act (No. 33 of 2005). Nurses are also members of society. Therefore, they have a moral obligation to serve their patients according to society's expectations, which often decides what constitutes right and wrong. Nurses thus have an obligation and moral duty to render quality and safe nursing care.

In addition to providing quality care to patients in a safe environment, a nurse's role also entails patient advocacy—helping patients understand their medical conditions and making informed ethical decisions in that context. In this context, ethical dilemmas —albeit from two varying but interrelated stakeholder categories and interests—conjoin the extent of decision‐making by both nurses, on the one hand, and patients, on the other (Desai et al., 2020). Ethical principles guide nurses' decision‐making in the event of a dilemma. In addition to their ethical principles and the Patients' Rights Charter, nurses in South Africa are also bound by morals and societal expectations, such as the African philosophy of Ubuntu. Therefore, ethical principles such as respect for autonomy, beneficence, non‐maleficence, justice, and the philosophy of Ubuntu guide nurses in ethical decision‐making.

## Respect for autonomy

The principle of respect for autonomy is premised on respecting other's rights to dignity and their ability and freedom to make informed choices and decisions. Accordingly, nurses must exercise autonomous decision‐making regarding their obligations and right to serve vis‐a‐vis their right to life, which is endangered by situations of PPE shortages amid the COVID‐19 pandemic. Respect for autonomy also entails informed consent (Owonikoko, 2013). Nurses have an unwritten consent with patients, backed by the professional, legal, and ethical/moral codes of practice (for nurses) balanced with a patients' repertoire of rights. In mediating through the milieu of unwritten consent, and exercising their right to autonomous decision‐making, nurses are not expected to discriminate against patients based on a diagnosis while treating and caring for them, notwithstanding the possible threats and risks to their own lives.

## Beneficence and non‐maleficence

In the context of this paper, beneficence premises on nurses doing good to others, their patients, while non‐maleficence concerns the avoidance or prevention of harm. In the case of the COVID‐19 pandemic, nurses are expected to treat and care for their patients and promote beneficence and non‐maleficence; meanwhile, employers are obligated to provide a safe environment through the provision of PPE (Department of Labor, 2004). In cases where the employer does not provide PPE as legally required by COVID‐19 prescripts, both the nurses' and patients' lives are in jeopardy. Such a situation renders nurses' beneficence difficult because the decision to “do good” entails the treatment and care to clients while endangering one's life and the lives of patients, colleagues, own family, and significant others—such as those met in public transport. It then becomes the responsibility of the nurse to weigh the harm and to do good benefits.

Nurses in South Africa are also bound by the Ubuntu philosophy, an African ethic, cognate from the idiom, “*Umuntu ngumuntu nga bantu*.” Translated literally, this means “A person is a person through other persons” (Mulaudzi & Peu, 2014; Mulaudzi et al., 2009), or “I am, because we are” (Metz, 2019). The core values embedded in Ubuntu, such as mutual respect, humanness, trust, honesty, cohesiveness, and solidarity, are commensurate with ethical principles that guide the nursing practice (Mulaudzi et al., 2018). The Ubuntu context provides a perspective to analyze the right to strike action regarding personal and community right‐doing (Mangena, 2016; Sambala et al., 2020). Ubuntu offers a starting point for negotiating the common good and social aspects of doing good. Ubuntu emphasizes values such as respect, reciprocity, relationship, and solidarity.

The Ubuntu philosophy essentializes the virtues of respect because a person can only see the other through the value they allocate to respect. In the COVID‐19 context, nurses demonstrate their respect by wearing PPE to protect themselves and their patients to avoid further spread of the infection. Similarly, the State is obliged to reciprocate the respect shown by nurses and the value they bring to both the healthcare system and the nursing profession.

Solidarity and cohesion are two critical Ubuntu principles with a significant bearing on nursing ethics. Cohesion exists in the context of *how* society views and supports each other from the perspective of the other (Sambala et al., 2020). The public's opinion of the nursing profession during the COVID‐19 pandemic impacts nurses' decision‐making regarding their exercise of the right to protest or not. Conversely, the decision by nurses not to protest also affects the degree or extent of solidarity and patient advocacy in respect of providing and receiving essential treatment and care services in a safe environment (Desai et al., 2020). Rather than opt for (protected or unprotected) strike action during the COVID‐19 pandemic, nurses resorted to expressing their PPE‐related grievances through the media. Due to the public's view of the rationality and justification of the non‐strike actions by nurses during the COVID‐19 pandemic, they subsequently generated and received overwhelming public support and solidarity (Tuohey, 2007). This public response is based on the value of respect for the common good and cohesive unity of purpose between themselves (the public) and the nurses (who are also members of society). Such a state of affairs epitomizes the value and respect that members of the same group(s) allocate to dialogue as a pivotal aspect of decision‐making by consensus for the common good (Tuohey, 2007).

### Justice

Justice entails equity, fairness, and proportionality. State healthcare entities need to ensure that PPE is fairly distributed between different hospitals and categories of health workers. Nurses and doctors must receive the same PPE if they work in the same wards (Tang et al., 2020). Hospitals in rural areas must receive the same PPE as hospitals in urban areas and special designated COVID‐19 hospitals. Binkley and Kemp (2020) contend that hospitals and healthcare systems should develop transparent, collaborative, and fair PPE rationing and allocation policies. The WHO stipulates unequivocally that as first responders, nurses and doctors working directly with COVID‐19 patients require N95 masks, while those in low‐risk wards use surgical masks (WHO, 2020). On this premise, the Center for Disease Control (CDC) issued guidelines to optimize the supply and distribution of N95 masks to address PPE shortages induced by pressures on the global supply chain management systems during the coronavirus pandemic (Cdcgov, 2020).

The South African justice system recognizes the principles of fairness, equity, and proportionality. These principles are embedded in the South African Constitution (Act no. 108 of 1996). Everyone has the right to fair labor practice in terms of Section 23 of the Constitution. If labor practices are not fair, workers are given the right to strike. The right to embark on strike action is embedded in Section 23(2)(c) of the Constitution, which provides that “Every worker has the right to strike” (Constitution of the Republic of South Africa, 1996). In essence, a strike is then emblematic of workers' withdrawal of their labor to bargain with employers on aspects of their employer–employee relations (Currie & De Waal, 2016). On numerous occasions, the courts have stressed the importance of workers' right to strike action. For instance, in *NUMSA v. Bader Bop*, the court stated that the right to strike is essential to workers' right to dignity and is a mechanism used by workers to enforce their rights (National Union of Metal Workers of South Africa and Others v. Bader Bop (Pty) Ltd. and Another (CCT14/02) (2002) ZACC 30).

Nurses have the right to a safe working environment and working conditions consistent with the rights to equality and dignity. Together, these rights should neither be negated nor de‐emphasized by the presence of a pandemic. The absence of PPE in the context of the COVID‐19 pandemic has life‐threatening consequences for nurses (Chaib, 2020) and negates their fundamental human right to work in a healthy environment (Constitution of the Republic of South Africa, 1996). It is within this context that the right to strike action and justice‐based ethics should be understood. The paragraphs below discuss the constitutional and justice‐based approach to the decision to strike.

### Right to strike in the South African constitution

The right to strike action is an unqualified right in the Constitution, which means it is inviolable and does not have any qualifications or limitations attached to it. Notwithstanding the absence of any direct constitutional limitations or qualifications, Section 65, read with section 71 of the LRA (Act No. 66 of 1995), places administrative and substantive limitations on the right to strike action by workers providing essential services. Under their provision of services classified as essential workers in this employment category, they are legally prevented from engaging in strike action. Section 213 of the LRA defines essential services as, “A service, the interruption of which endangers the life, personal safety or health of the whole or any part of the population.” Consequently, nurses were declared as providers of essential services in 1998 by the Essential Services Committee, which means that nurses cannot participate in protected strikes under the LRA (GNR.1216 of 12 September 1997).

The limitation placed by the LRA on the right of nurses as essential services workers to strike has not yet been tested against Section 36 of the Constitution. The latter stipulates that a right in the Bill of Rights may be limited in terms of the law of general application, provided that the limitation is reasonable and justifiable. The Section 36 test requires that the limitation of rights should consider factors such as the nature of the right; the importance of the purpose of the limitation; the nature and extent of the limitation; the relationship between the limitation and its purpose; and whether there are less restrictive means to achieve the intended purpose.

The COVID‐19 pandemic presents a unique situation that could nullify the assumption that Section 65 and Section 71 limitation of nurses’ right to strike in the LRA withstands the limitations test. The State's failure to provide PPE and the subsequent need for strike action by nurses cannot be understood purely as a case of the right of nurses to strike vis‐a‐vis the right of patients to healthcare. It is trite that rights need to be interpreted and understood in clusters and within a particular context. In this case, the right of nurses to strike against unfair working conditions due to the absence of PPE is linked to the right to life and the right to dignity of both the nurses and the patients. When interpreting the limitation established by Section 71 and Section 65 of LRA, the question that follows is: “*What happens in a case where one person's right to life must be weighed against another's right to life?*”

The Section 36 test is two‐fold. First, one should determine whether the impugned law limits the right in question (limitation). Second, one should determine whether the limitation of the right is justifiable (justifiability). The section below presents a brief Section 36 analysis to further articulate the rights‐based approach in the context of the right to strike by nurses as essential services workers.

### Limitations

Part one of the limitations test requires an analysis of the scope and content of the right to strike, as well as the meaning and effect of essential services to determine whether or not the essential services provisions do limit the right to strike (Ex Parte Minister of Safety and Security and Others: In Re S v. Walters and Another (CCT28/01) (2002) ZACC 6). The ILO defines the right to strike as “a means by which workers and their associations may legitimately promote and defend their economic and social interests” (Gernigon et al., 2000, p. 12). The ICN defines strike action as “cessation of work or a refusal to work or continue to work for the purpose of compelling an employer to agree to conditions of work which could not be achieved through negotiation” (ICN, 2011).

Although the right to strike itself does not entail any internal qualifiers, the LRA sets out several procedural and substantive limitations to the right. Section 65(1)(d)(i) of the LRA prevents any essential services worker from taking part in strike action. The ILO also recognizes the limitation placed by the right to strike on essential services (Novitz, 2016). In this regard, the ILO's definition of essential services is the same as the LRA's in that it recognizes the hospital sector as an essential service. The exact designation or meaning of “essential” depends on each country's circumstances (Gernigon et al., 2000). The ILO further recognizes the limitation on the right to strike in emergencies (Gernigon et al., 2000).

The limitation prevents essential services workers from exercising the right to strike in its entirety and presents a different set of collective bargaining rules that exclude the right to strike (Subramanien & Joseph, 2019). In such a context, the question that follows and is of particular importance should be: “*Is the limitation reasonable and justifiable?*”

### Justification analysis

The justification analysis of Section 36 of the Constitution interrogates the extent to which competing interests could be balanced (*Johncom Media Investments Limited v. M and Others* (CCT 08/08) (2009) ZACC 5; (2009) (4) SA 7 (CC)). Such analysis requires a “weighing‐up of the nature and importance of the rights that are limited together with the extent of the limitation as against the importance and purpose of the limiting enactment” (*Ex Parte Minister of Safety and Security and Others: In Re S v. Walters and Another* (CCT28/01) (2002) ZACC 6). This weighing‐up concedes to a “global judgment on [the] proportionality” of the limitation (*S v. Bhulwana, S v. Gwadiso* (CCT12/95, CCT11/95) [1995] ZACC 11). Essentially, the LRA's Section 71 limitation on the right to strike accentuates the question of balancing the competing rights in the Bill of Rights. On the one hand, there is the right to strike, read together with the right to life and bodily integrity and health of nurses; both of which rights would be at risk without PPE and subsequently infringe on patients' right to a safe working environment. On the other hand, there is the right to health, the right to dignity, and patients' right to life. All of these can be adversely affected on a large scale if nurses undertake strike action.

Strike action is generally understood within the context of socio‐economic liberation (van Rensburg & van Rensburg, 2013). Labor strikes are usually intended to advance employee rights and interests regarding wage or salary negotiations and other unfair or exploitative labor practices in the ambit of socio‐economic interests (van Rensburg & van Rensburg, 2013). In this case, however, the right to strike is contextualized within the occupational health and safety environment. If not corrected, the attendant legal, ethical/moral and professional contradictions could lead to the loss of life of both nurses and patients if not corrected. Some nurses may fear for their lives working without PPE (which is a health and safety concern) but may not have the financial luxury to partake in unprotected strike action at the risk of loss of employment or salary (which is a socio‐economic concern).

Essential services are clearly defined and are integrally vital during a health crisis such as COVID‐19. There is a clear need for a process that limits the right to strike for nurses. If nurses strike, there is a real risk to the lives of many. Section 231 includes a clear definition of essential services, and there is no doubt that nurses fit into this category. The purpose of the essential services provision is also clarified by the definition of such services, in the context of which strike action could adversely affect the lives of the public. The 2010 public service strike evinces the possible loss of lives resulting from strike action by nurses. The strike led to dire consequences and affected access to medication, unnecessary movement of patients, and other issues (van Rensburg & van Rensburg, 2013). In this case, this would be on an even larger scale.

On the other hand, the limitation established by the essential services provisions does not consider the fact that the right to strike in this case ought to be understood in the context of the right to life of nurses. Statistical evidence on the dangers and risks posed by COVID‐19 shows that nurses increasingly confront the risk of death, notwithstanding PPE provision. Without PPE, the lives of nurses and those exposed to them are at an increased risk. The right to life is unqualified and is one of the most fundamental human rights (*State v. Makwanyane*, 1995). Of particular interest is that strike action imperils the lives of patients, and a limitation on those rights places the lives of nurses at risk and—by implication—the lives of the patients they treat. In this regard, the balancing exercise is between the loss of lives for both parties. What happens if two lives are weighed against each other? How do the courts determine which life holds more value?

A critical look at the South African Patients’ Bill of Rights suggests that nurses' right to strike is justifiable. The first right on the South Africa Patients' Bill of Rights states that every patient has the right to a safe and healthy environment. The safety and quality of patient care are determined by the environment in which care is provided (Singh & Mathuray, 2018). Unfortunately, the inability of the nurses to provide quality nursing care is often due to a lack of resources beyond their jurisdiction and the State and other healthcare institutions need to provide appropriate resources.

When balanced against nurses' right to strike, the right to life, health, and the human dignity of patients are reasonable and justifiable, provided the right to strike is viewed in a silo. However, it could be argued that the failure to provide PPE infringes on both the patients' right to a safe environment and nurses' rights to life, health, and dignity. In such a context (failure of PPE provision), strike action by nurses is presented as justifiable. However, undertaking a balancing exercise in that context could project the limitation as both unreasonable and unjustifiable.

The justifiability of the argument supporting nurses’ strike action based on the lack of PPE may not be a viable recourse for nurses and healthcare facilities in the current COVID‐19 environment. Such an eventuality or perceived recourse would diminish hospitals' nursing human resources capacity in the middle of such a devastating pandemic. In the past, replacement staff was appointed to fill the gaps for striking nurses (van Rensburg & van Rensburg, 2013). The growing number of COVID‐19 cases has necessitated the maximum provision of essential hospital services without any disruptions, and this may not be a luxury that the State can afford at this time.

The discussion above has presented a rights‐based approach to nurses’ strike action. We find that there may be arguments supporting a protected strike in terms of the LRA stipulations. In South Africa, an unprotected strike action remains a possibility and is not criminalized. Considering the innocuous legal implications of an unprotected strike with the risk to the lives of nurses, the question then becomes: What are the ethical considerations of strike action in a pandemic by both the nurse (as unionized employee) and the State (as a nurse employer through the National Department of Health)? It must also be borne in mind that nurses have professional and ethical obligations always to put the patients' interests ahead of theirs.

## CONCLUSION

The questions this paper sought to answer are complex. Taking into consideration the cornerstone of nurses’ ethical obligation to always put patients first, *what ethical duty do these nurses have to work in the event the State does not provide PPE to all nurses working on COVID‐19 pandemic when the work poses a genuine threat to their lives and that of their patients? Would it be unethical for nurses to decide to preserve their lives over the lives of their patients?*

The discussion presented in this paper seeks to accentuate the conversation concerning ethical principles in strike action. This is not the first paper in the latter regard. However, it is unique in that it highlights the issue against the backdrop of a global pandemic. It is also the first to interrogate strike action in the context of direct risks posed to the lives of nurses whose duty is to place patient care above all else.

In terms of the human rights approach, there are valid grounds supporting arguments for a rethinking of limitations about nurses' right to strike in contexts where their lives are significantly at risk. This paper does not make a conclusion on the dilemma posed by limitations on nurses' right to strike action in a COVID‐19 milieu. It is an issue whose finalization rests within the jurisdiction or purview of the courts of law. What we do find, however, is that a balancing exercise on the right to life is complex, and the answers are neither obvious nor simple. The law is clear. The human rights framework does not provide for strike action for nurses because such action could compromise the essential services necessary to save the lives of the population. Against such a backdrop, this paper interrogates the possibility of making a human rights‐based argument in support of an exception to the rule. Accordingly, we find that a legal argument could be advanced on the provision that the right to strike is understood within the context of the right to life for nurses and their patients. We further find that without court action, protected strike action is not an option for nurses, and unprotected strike action remains an option and a possibility only when PPE provision is not made a reality. Nurses threatening strike action may find that the consequences of an unprotected strike may be worth the risk in highly exceptional situations.

The absence of PPE in a pandemic is not an issue to be taken lightly. The ramifications are grave for patients, nurses, and those living with them. In the practical sense, compelling nurses to work without PPE induces greater risks of infection and death to patients as well. Hospitals are also confronted with the risk of losing nursing personnel, forced to leave work to recover or even lose their lives to the disease.

In the context of this paper, the choice or decision to strike is reflective of a desperate call for PPE, whose purpose would be to protect the lives of both nurses and patients. It would be a mistake to understand this as a patient‐versus‐nurse issue. It would be another mistake to simply understand this as a one life‐versus‐another life issue. The ripple effect caused by the absence of PPE is detrimental to both patient care and the health of nurses (Huang et al., 2020, Jecker et al., 2020). Inadvertently, the absence of PPE turns nurses into patients*. If the ethical duty is to patients, what of the protection of nurses who become patients? If nurses themselves become patients, who will be at the frontlines of the COVID‐19 pandemic?*

## AUTHOR CONTRIBUTION

FM conceptualized the paper and wrote on the ethical aspect of the paper and reviewed literature. MM wrote the legal aspect of the paper and reviewed literature. RA wrote the introduction and the abstract and searched and reviewed literature and put together bibliography list. MDM wrote the ethical aspect of the paper and reviewed literature

## References

[inr12703-bib-0001] American Nurses Association (2020) Nurses, ethics and the response to the COVID‐19 pandemic. Available at: nurses‐ethics‐and‐the‐response‐to‐the‐covid‐19‐pandemic. https://www.nursingworld.org/~4981cc/globalassets/covid19/nurses-ethics-and-the-response-to-the-covid-19-pandemic_pdf-1.pdf (Accessed 30 June 2021)

[inr12703-bib-0002] ANA, E.A.B. (2019) ANA Position Statement: The ethical responsibility to manage pain and the suffering it causes. The Online Journal of Issues of Nursing, 24(1).

[inr12703-bib-0003] Binkley, C.E. & Kemp, D.S. (2020) Ethical rationing of personal protective equipment to minimize moral residue during the COVID‐19 pandemic. Journal of the American College of Surgeons, 230(6), 1111–1113. 10.1016/j.jamcollsurg.2020.03.031 32278727 PMC7195046

[inr12703-bib-0004] Center for Disease Control and Prevention (2020) Strategies for optimizing the supply of N95 respirators. Updated 29 February 2020. Available at: https://www.cdc.gov/coronavirus/2019-ncov/hcp/respirators-strategy/index.html (Accessed 1 June 2020)

[inr12703-bib-0005] Chaib, F. (2020) Shortage of personal protective equipment endangering health workers worldwide. Available at: https://www.who.int/news-room/detail/03-03-2020-shortage-of-personal-protective-equipment-endangering-health-workers- worldwide (Accessed 30 May 2020)

[inr12703-bib-0006] Chersich, M.F. , Gray, G. , Fairlie, L. , Eichbaum, Q. , Mayhew, S. , Allwood, B. , English, R. , Scorgie, F. , Luchters, S. , Simpson, G. , Haghighi, M.M. , Pham, M.D. & Rees, H. (2020) COVID‐19 in Africa: care and protection for frontline healthcare workers. Globalization and Health, 16(1), 46. 10.1186/s12992-020-00574-3 32414379 PMC7227172

[inr12703-bib-0007] Chima, S.C. (2013) Global medicine: Is it ethical or morally justifiable for doctors and other healthcare workers to go on strike? BMC Medical Ethics, 14(1), S5. 10.1186/1472-6939-14-S1-S5 24564968 PMC3878318

[inr12703-bib-0008] Cucinotta, D. & Vanelli, M. (2020) WHO declares COVID‐19 a pandemic. Acta Bio‐medica: Atenei Parmensis, 91(1), 157–160. 10.23750/abm.v91i1.9397 32191675 PMC7569573

[inr12703-bib-0009] Currie, I. & De Waal, J. (2016) The bill of rights handbook. Cape Town: Juta and Company Ltd.

[inr12703-bib-0010] Daniels, R.C. (2020) Factors influencing safe patient care provided by professional nurses in a private healthcare organisation in the Western Cape. Stellenbosch: Stellenbosch University.

[inr12703-bib-0011] Department of Health, D. (2020) Department of health: Directions to address, prevent and combat the spread of COVID‐19: Repealed. *. Government gazette no. 43217*. Available at: https://openbylaws.org.za/za/act/gn/2020/457/eng/resources/eng.pdf (Accessed 6 July 2021).

[inr12703-bib-0012] Department of Labour . (1993) Occupational Health and Safety Amendment Act (no. 181 of 1993). Pretoria: Government Printers.

[inr12703-bib-0013] Department of Labour . (2004) Personal protective equipment: Eyes and face protection. Occupational Safety and Health Administration, 5(1), 8–16. 10.5923/j.safety.20160501.02

[inr12703-bib-0014] Desai, A. , Lankford, C. & Schwartz, J. (2020) With crisis comes opportunity: Building ethical competencies in light of COVID‐19. Ethics & Behavior, 30(6), 401–413. 10.1080/10508422.2020.1762603

[inr12703-bib-0015] Dowie, I. (2017) Legal, ethical and professional aspects of duty of care for nurses. Nursing Standard, 32(16‐19), 47–52. 10.7748/ns.2017.e10959 29250939

[inr12703-bib-0016] Dzinamarira, T. , Nachipo, B. , Phiri, B. & Musuka, G. (2021) COVID‐19 vaccine roll‐out in South Africa and Zimbabwe: Urgent need to address community preparedness, fears and hesitancy. Vaccines, 9(3), 250. 10.3390/vaccines9030250 33809002 PMC8000117

[inr12703-bib-0017] (2002) Ex Parte Minister of Safety and Security and Others: In Re S v. Walters and Another (CCT28/01) [2002]ZACC 6).

[inr12703-bib-0018] Fisher, M.T. (2017) An exploration into student nurses' perception of patient safety and experience of raising concerns. Ann Arbor: University of Northumbria at Newcastle (United Kingdom).

[inr12703-bib-0019] Gernigon, B. , Odero, A. & Guido, H. (2000) ILO principles concerning the right to strike. Geneva: International Labour Organization.

[inr12703-bib-0020] Gilbert, M. , Pullano, G. , Pinotti, F. , Valdano, E. , Poletto, C. , Boëlle, P.‐Y. , D'Ortenzio, E. , Yazdanpanah, Y. , Eholie, S.P. , Altmann, M. , Gutierrez, B. , Kraemer, M.U. G. & Colizza, V. (2020) Preparedness and vulnerability of African countries against importations of COVID‐19: a modelling study. The Lancet, 395(10227), 871–877. 10.1016/S0140-6736(20)30411-6 PMC7159277 32087820

[inr12703-bib-0021] Gondi, S. , Beckman, A.L. , Deveau, N. , Raja, A.S. , Ranney, M.L. , Popkin, R. & He, S. (2020) Personal protective equipment needs in the USA during the COVID‐19 pandemic. The Lancet, 395(10237), e90–e91. 10.1016/s0140-6736(20)31038-2 PMC7255297 32416784

[inr12703-bib-0022] Haddad, L.M. & Geiger, R.A. (2020) Nursing ethical considerations. Treasure Island, FL: StatPearls Publishing. 30252310

[inr12703-bib-0023] Hall, V.J. , Foulkes, S. , Saei, A. , Andrews, N. , Oguti, B. , Charlett, A. , Wellington, E. , Stowe, J. , Gillson, N. , Atti, A. , Islam, J. , Karagiannis, I. , Munro, K. , Khawam, J. , Chand, M.A. , Brown, C.S. , Ramsay, M. , Lopez‐Bernal, J. , Hopkins, S. & Group, S.S. (2021) COVID‐19 vaccine coverage in health‐care workers in England and effectiveness of BNT162b2 mRNA vaccine against infection (SIREN): a prospective, multicentre, cohort study. Lancet (London, England), 397(10286), 1725–1735. 10.1016/S0140-6736(21)00790-X PMC8064668 33901423

[inr12703-bib-0024] Huang, L. , Lin, G. , Tang, L. , Yu, L. & Zhou, Z. (2020) Special attention to nurses’ protection during the COVID‐19 epidemic. Critical Care, 24(1), 120. 10.1186/s13054-020-2841-7 32220243 PMC7101882

[inr12703-bib-0025] ICN . (2011) Industrial action. Available at: https://www.icn.ch/sites/default/files/inline‐files/C11_Industrial_action.pdf (Accessed 6 July 2021).

[inr12703-bib-0026] Ip, V. , Özelsel, T.J.P. , Sondekoppam, R.V. & Tsui, B.C.H. (2020) COVID‐19 pandemic: Greater protection for health care providers in the hospital “hot zones”? Anesthesia and Analgesia, 131(1), e37–e38. 10.1213/ANE.0000000000004880 33035019 PMC7179064

[inr12703-bib-0027] Jecker, N.S. , Wightman, A.G. & Diekema, D.S. (2020) Prioritizing frontline workers during the COVID‐19 pandemic. American Journal of Bioethics, 20(7), 128–132. 10.1080/15265161.2020.1764140 32401170

[inr12703-bib-0028] (2009) *Johncom Media Investments Limited v. M and Others* (CCT 08/08) [2009]ZACC 5; 2009 (4) SA 7 (CC).

[inr12703-bib-0029] Kennedy, A. (2021) The International Council of Nurses in the time of the COVID‐19 pandemic. International Nursing Review, 68 (2), 144–146. 10.1111/inr.12681 34053077 PMC8207128

[inr12703-bib-1030] Liu, Q. , Zheng, Z. , Zheng, J. , Chen, Q. , Liu, G. , Chen, S. , Chu, B. , Zhu, H. , Akinwunmi, B. , Huang, J. , Zhang, C. J. P. & Ming, W.‐K. (2020) Health Communication Through News Media During the Early Stage of the COVID‐19 Outbreak in China: Digital Topic Modeling Approach. Journal of Medical Internet Research, 22(4), e19118. 10.2196/19118 32302966 PMC7189789

[inr12703-bib-0030] Leider, J. P. , DeBruin, D. , Reynolds, N. , Koch, A. & Seaberg, J. (2017) Ethical guidance for disaster response, specifically around crisis standards of care: A systematic review. American Journal of Public Health, 107(9), e1–e9. 10.2105/ajph.2017.303882 PMC5551597 28727521

[inr12703-bib-0031] Mangena, F. (2016) African ethics through Ubuntu: A postmodern exposition. Journal of Pan African Studies, 9(2), 66–80.

[inr12703-bib-0032] Mathe, T. (2020) Gauteng nurses say they did not take an oath to die. *Mail & Guardian*, 29 May. Available at: https://mg.co.za/coronavirus‐essentials/2020‐05‐29‐gauteng‐nurses‐say‐they‐did‐not‐take‐an‐oath‐to‐die/ (Accessed 30 May 2020).

[inr12703-bib-0033] McQuoid‐Mason, D.J. (2020) Is the COVID‐19 regulation that prohibits parental visits to their children who are patients in hospital invalid in terms of the Constitution? What should hospitals do? South African Medical Journal, 110(11), 1086–1087. 10.7196/samj.2020.v110i11.15273 33403983

[inr12703-bib-0034] Metz, T. (2019) *The African ethic of Ubuntu*. 1000‐Word philosophy: An introductory anthology. Available at: https://1000wordphilosophy.com/2019/09/08/the‐african‐ethic‐of‐ubuntu/ (Accessed 6 July 2021).

[inr12703-bib-0035] Mulaudzi, F. , Mogale, R. & Masoga, M. (2018) Revisiting current nursing ethics: Can Ubuntu foster an environment for ethics of care?. Trenton, New Jersey: Ubuntu and Personhood Africa World Press.

[inr12703-bib-0036] Mulaudzi, F.M. , Libster, M.M. & Phiri, S. (2009) Suggestions for creating a welcoming nursing community: Ubuntu, cultural diplomacy, and mentoring. International Journal of Human Caring, 13(2), 45–51. 10.20467/1091-5710.13.2.45

[inr12703-bib-0037] Mulaudzi, F.M. & Peu, M.D. (2014) Communal child‐rearing: The role of nurses in school health. Curationis, 37(1). 1–7. 10.4102/curationis.v37i1.1158 25685906

[inr12703-bib-0038] Murray, J.S. (2010) Moral courage in healthcare: Acting ethically even in the presence of risk. Online Journal of Issues in Nursing, 15(3).

[inr12703-bib-0039] Mwiandi, M. (2011) Leadership and the transformation of Society. Nairobi: East African Educational Publishers.

[inr12703-bib-0040] (2002) National Union of Metal Workers of South Africa and Others v. Bader Bop (Pty) Ltd and Another (CCT14/02) [2002]ZACC 30.

[inr12703-bib-0041] Novitz, T. (2016) The restricted freedom to strike: Far‐reaching ILO jurisprudence on the public sector and essential services. Comparative Labor Law and Policy Journal, 38(3), 353–374.

[inr12703-bib-0042] Oguisso, T. , Takashi, M.H. , Freitas, G.F.D. , Bonini, B.B. & Silva, T.A.D. (2019) First International Code of Ethics for Nurses. Texto & Contexto–Enfermagem, 28, e20180140. 10.1590/1980-265x-tce-2018-0140

[inr12703-bib-0043] Owonikoko, T.K. (2013) Upholding the principles of autonomy, beneficence, and justice in phase I clinical trials. The Oncologist, 18(3), 242–244. 10.1634/theoncologist.2013-0014 23457003 PMC3607517

[inr12703-bib-0044] Padilla, M. (2020) It feels like a war zone’: doctors and nurses plead for masks on social media. *The New York Times*, 19 March. Available at: https://www.nytimes.com/2020/03/19/us/hospitals‐coronavirus‐ppe‐shortage.html (Accessed 1 June 2020).

[inr12703-bib-0045] Page, K. (2012) The four principles: Can they be measured and do they predict ethical decision making? BMC Medical Ethics, 13(1), 10. 10.1186/1472-6939-13-10 PMC3528420 22606995

[inr12703-bib-0046] The Republic of South Africa . (1996) Constitution of the Republic of South Africa (Act No. 108 of 1996. Pretoria: Government Printers.

[inr12703-bib-0047] Roelf, W. & Winning, A. (2020) South African union takes govt to court over COVID‐19 gear shortage. *Reuters*, 7 April. Available at: https://www.reuters.com/article/ozabs-uk-health-coronavirus-safrica-idAFKBN21P269-OZABS (Accessed 30 May 2020).

[inr12703-bib-0048] Roshanzadeh, M. , Vanaki, Z. & Sadooghiasl, A. (2020) Sensitivity in ethical decision‐making: The experiences of nurse managers. Nursing Ethics, 27(5), 1174–1186. 10.1177/0969733019864146 31462175

[inr12703-bib-0049] Rowan, N.J. & Laffey, J.G. (2020) Challenges and solutions for addressing critical shortage of supply chain for personal and protective equipment (PPE) arising from Coronavirus disease (COVID19) pandemic–Case study from the Republic of Ireland. Science of the Total Environment, 725, 138532. 10.1016/j.scitotenv.2020.138532 PMC7195029 32304970

[inr12703-bib-0050] Royal College of Nursing (2020) Joint letter to British Safety Industry Federation on personal protective equipment for male and female users. Available at: https://www.rcn.org.uk/covid-19/rcn-open-letters (Accessed 1st June 2020)

[inr12703-bib-0051] Sambala, E.Z. , Cooper, S. & Manderson, L. (2020) Ubuntu as a framework for ethical decision making in Africa: Responding to epidemics. Ethics & Behavior, 30(1), 1–13. 10.1080/10508422.2019.1583565

[inr12703-bib-0052] Singh, A. & Mathuray, M. (2018) The nursing profession in South Africa—Are nurses adequately informed about the law and their legal responsibilities when administering health care? De Jure, 51(1). 122–139. 10.17159/2225-7160/2018/v51n1a8

[inr12703-bib-0053] Subramanien, D. & Joseph, J. (2019) The right to strike under the Labour Relations Act 66 of 1995 (LRA) and possible factors for consideration that would promote the objectives of the LRA. Potchefstroom Electronic Law Journal (PELJ), 22, 1–39. 10.17159/1727-3781/2019/v22i0a4400

[inr12703-bib-0054] *State v. Makwanyane and Mchunu (1995)* CCT/3/94

[inr12703-bib-0055] *State v. Bhulwana* , *S v. Gwadiso* (1995) (CCT12/95, CCT11/95) [1995]ZACC 11

[inr12703-bib-0056] Tang, L.‐H. , Tang, S. , Chen, X.‐L. , Zhang, S. , Xiong, Y. , Chen, R. , Li, W. , Liu, H.‐M. , Xia, Z.‐Y. & Meng, Q.‐T. (2020) Avoiding health worker infection and containing the coronavirus disease 2019 pandemic: Perspectives from the frontline in Wuhan. International Journal of Surgery, 79, 120–124. 10.1016/j.ijsu.2020.05.060 32454250 PMC7245215

[inr12703-bib-0057] Tuohey, J.F. (2007) A matrix for ethical decision making in a pandemic. The Oregon tool for emergency preparedness. Health Progress, 88(6), 20–25 18062370

[inr12703-bib-0058] van Rensburg, A.J. & van Rensburg, D.J. (2013) Nurses, industrial action and ethics: Considerations from the 2010 South African public‐sector strike. Nursing Ethics, 20(7), 819–837. 10.1177/0969733012473771 23454981

[inr12703-bib-0059] WHO. (2020) Rational use of personal protective equipment for coronavirus disease (COVID‐19) and considerations during severe shortages: interim guidance, 6 April 2020. World Health Organization. https://apps.who.int/iris/handle/10665/331695 (Accessed 30 May 2020)

[inr12703-bib-0060] WHO. (2021) WHO Coronavirus (COVID‐19) dashboard. World Health Organization. Available at: https://covid19.who.int/ (Accessed 6 July 2021).

[inr12703-bib-0061] Zhang, S.X. , Marioli, F.A. & Gao, R. (2021) A second wave? What do people mean by COVID Waves?–A working definition of epidemic waves. medRxiv: the preprint server for health sciences. 10.1101/2021.02.21.21252147 PMC8448159 34548826

